# Fine-Tuning ER Stress Signal Transducers to Treat Amyotrophic Lateral Sclerosis

**DOI:** 10.3389/fnmol.2017.00216

**Published:** 2017-07-05

**Authors:** Danilo B. Medinas, Jose V. González, Paulina Falcon, Claudio Hetz

**Affiliations:** ^1^Program of Cellular and Molecular Biology, Center for Molecular Studies of the Cell, Institute of Biomedical Sciences, University of ChileSantiago, Chile; ^2^Faculty of Medicine, Biomedical Neuroscience Institute, University of ChileSantiago, Chile; ^3^Center for Geroscience, Brain Health and MetabolismSantiago, Chile; ^4^Buck Institute for Research on AgingNovato, CA, United States; ^5^Department of Immunology and Infectious Diseases, Harvard School of Public HealthBoston, MA, United States

**Keywords:** ALS, ER stress, UPR, IRE1α, protein aggregation

## Abstract

Amyotrophic lateral sclerosis (ALS) is a fatal neurodegenerative disease characterized by the progressive loss of motoneurons and paralysis. The mechanisms underlying neuronal degeneration in ALS are starting to be elucidated, highlighting disturbances in motoneuron proteostasis. Endoplasmic reticulum (ER) stress has emerged as an early pathogenic event underlying motoneuron vulnerability and denervation in ALS. Maintenance of ER proteostasis is controlled by a dynamic signaling network known as the unfolded protein response (UPR). Inositol-requiring enzyme 1 (IRE1) is an ER-located kinase and endoribonuclease that operates as a major ER stress transducer, mediating the establishment of adaptive and pro-apoptotic programs. Here we discuss current evidence supporting the role of ER stress in motoneuron demise in ALS and build the rational to target IRE1 to ameliorate neurodegeneration.

## Introduction

Amyotrophic lateral sclerosis (ALS) is a fatal neurodegenerative disease that affects motoneurons of cerebral cortex, brainstem and spinal cord, leading to muscle weakness, paralysis and premature death within 3–5 years after diagnosis (Turner et al., 2013; Peters et al., 2015). Besides motoneuron loss, the intracellular accumulation of protein inclusions of different compositions is a hallmark of ALS (Turner et al., 2013; Peters et al., 2015; Ruegsegger and Saxena, 2016). The hereditary forms of the disease account for approximately 5%–10% of total cases and are termed familial ALS (fALS), caused by mutations in different genes such as *SOD1*, *TARDBP*, *FUS* and hexanucleotide repeat expansions in *C9orf72* (Turner et al., 2013; Leblond et al., 2014; Peters et al., 2015). Interestingly, the corresponding mutant proteins and (repeat-associated non-ATG translated, RAN) dipeptides form protein oligomers and aggregates, leading to impaired proteostasis with resultant motoneuron dysfunction and death (Turner et al., 2013; Peters et al., 2015; Ruegsegger and Saxena, 2016). In sporadic cases of ALS (sALS), misfolding and aggregation of the same proteins in the absence of mutations suggest common pathogenic mechanisms in fALS and sALS (Neumann et al., 2006; Bosco et al., 2010; Farg et al., 2012).

The development of genetic models of ALS has enabled dissection of disease course at histological, cellular and molecular levels (Philips and Rothstein, 2015). Although multiple mechanisms are proposed to drive ALS (Taylor et al., 2016), several recent unbiased studies in mutant SOD1 transgenic mice and induced pluripotent stem cell (iPSC)-derived patient motoneurons have identified endoplasmic reticulum (ER) stress as an early and transversal pathogenic mechanism underlying selective vulnerability of motoneurons in ALS (Saxena et al., 2009; Kiskinis et al., 2014; Filézac de L’Etang et al., 2015; Sun et al., 2015). ER stress is a condition generated by abnormal levels of misfolded proteins in the ER lumen, engaging a signal transduction pathway termed the unfolded protein response (UPR). The UPR operates as a central controller of cell fate, mediating initial adaptive responses to restore proteostasis through various mechanisms including transcriptional and translational regulation, enhancement of protein quality control mechanisms, degradation of abnormal proteins, among other outputs (Hetz, 2012). The UPR is a binary pathway that shifts its signaling toward a terminal phase to eliminate irreversibly damaged cells through apoptosis (Walter and Ron, 2011). The adaptive UPR is marked by rapid inhibition of protein translation due to the phosphorylation of the eukaryotic initiation factor 2α (eIF2α), in addition to transcriptional induction of chaperones, foldases, protein quality control and degradation systems, lipid biosynthesis, among others. Under pathological conditions of chronic ER stress as observed in numerous neurodegenerative diseases (Hetz and Mollereau, 2014; Scheper and Hoozemans, 2015; Smith and Mallucci, 2016), the terminal UPR engages pro-inflammatory and apoptotic cascades leading to cell death (Urra et al., 2013; Oakes and Papa, 2015).

## UPR Signaling Pathways

**Figure 1:**
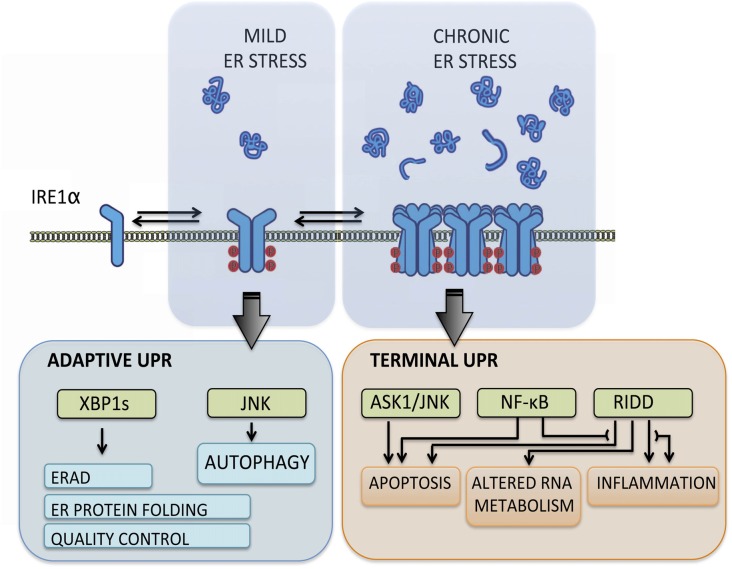
Inositol-requiringenzyme 1 (IRE1) signaling outputs. Under transient and mild endoplasmic reticulum (ER) stress, IRE1 undergoes dimerization and auto-transphosphorylation activating RNase activity and production of the potent transcription factor spliced X-box binding protein 1 (XBP1s), which induces adaptive programs to reduce protein misfolding, mediated by the upregulation of genes involved in protein folding, quality control, ER-associated protein degradation (ERAD) components and lipid biosynthesis. When ER stress is irremediable and chronic, IRE1 assembles into a scaffold platform for activation of ASK1-JNK and NF-κB pathways, which cause induction of apoptosis and modulates inflammation and autophagy levels. Furthermore, IRE1 overactivation decreases its RNase specificity and induces Regulated IRE1-dependent Decay (RIDD) activity, which degrades mRNA and microRNA and contributes to cell demise by depleting ER components and inducing pro-inflammatory and apoptotic factors.

The UPR transduces information about protein folding status from ER lumen to cytosol and nucleus through the action of various type-I ER transmembrane proteins that respond to the accumulation of misfolded proteins. These sensors reprogram the transcriptional and translational profile of the cell by a concerted action of transcription factors, phosphorylation events and RNA processing (Hetz et al., 2015). The mammalian UPR relies on three stress transducers, named activating transcription factor 6 (ATF6), protein kinase R (PKR)-like ER kinase (PERK) and inositol-requiring enzyme 1 (IRE1), being IRE1 the most conserved sensor from yeast to human (Wang and Kaufman, 2016). IRE1 is a kinase and endoribonuclease that upon ER stress is activated by dimerization and auto-transphosphorylation to catalyze the unconventional splicing of X-box binding protein 1 (XBP1) mRNA (Figure 1), thus leading to production of a potent transcription factor termed XBP1s (Hetz et al., 2015). During the adaptive UPR, XBP1s induces expression of ER chaperones and co-factors, ER-associated protein degradation (ERAD) components and lipid biosynthesis to increase the protein folding and quality control capacity (Walter and Ron, 2011). When ER stress is chronic, IRE1 is overactivated through assembly into high-order oligomers and reduces its substrate specificity to catalyze degradation of mRNA and microRNAs (Figure 1), an activity termed Regulated IRE1-dependent Decay (RIDD; Maurel et al., 2014). The activation of RIDD depletes ER components and reflects the terminal UPR directing cell fate towards apoptosis by directly controlling the stability of microRNAs, apoptosis genes and pro-inflammatory factors (Hollien and Weissman, 2006; Han et al., 2009; Hollien et al., 2009; Lerner et al., 2012; Ghosh et al., 2014). Furthermore, IRE1 can interact with cytosolic components, including adaptor proteins, to fine-tune UPR outputs in a dynamic fashion (Figure 1), comprising a protein platform termed “UPRosome” (Hetz and Glimcher, 2009). For instance, IRE1 can be coupled to JNK and NF-κB pathways through adaptor proteins to induce apoptosis upon prolonged ER stress (Urano et al., 2000; Hu et al., 2006). Thus, IRE1 signaling governs adjustment of proteostasis through XBP1-dependent transcriptional control, turning into a pro-degenerative effector when proteostasis cannot be recovered, engaging a variety of downstream pro-inflammatory and apoptotic regulators (Figure 1).

## The ER Folding Network and ALS

The involvement of ER stress on ALS pathogenesis has been inferred from multiple studies in patient post-mortem tissue and iPSC-derived motoneurons, as well as animal models of disease (see examples in Atkin et al., 2006, 2008; Ilieva et al., 2007; Hetz et al., 2009; Ito et al., 2009; Saxena et al., 2009; Sasaki, 2010; Matus et al., 2013; Kiskinis et al., 2014). A landmark study developed a comparative gene expression profiling of vulnerable and resistant motoneurons, identifying ER stress as the earliest pathological event in mutant SOD1 mice occurring before any denervation is detected (Saxena et al., 2009). On a recent follow-up study, the Saxena’s group reported altered ER chaperone network underlying differential susceptibility of motoneurons in ALS (Filézac de L’Etang et al., 2015). Briefly, the BiP co-chaperone SIL1 was found enriched in resistant while progressively reduced in vulnerable motoneurons over disease course, and SIL1 overexpression using adeno-associated virus was proven to be neuroprotective (Filézac de L’Etang et al., 2015). Using ribosome profiling of motoneurons and glia *in vivo*, the Cleveland’s group indicated that ER stress is a major pathological signature of motoneurons, and may mediate cell autonomous neurodegeneration cascades in mutant SOD1 models (Sun et al., 2015). Additional findings support the concept that motoneurons are selectively vulnerable to perturbations to ER function. For instance, deletion of one calreticulin allele, an essential ER chaperone, led to exacerbated muscle weakness and denervation in the mutant SOD1 mouse model, accelerating the progression of the disease (Bernard-Marissal et al., 2012). Importantly, motoneuron dysfunction due to deficiency of calreticulin did not involve increased motoneuron loss, suggesting a role of ER chaperone network at early stages of ALS leading to muscle denervation (Bernard-Marissal et al., 2012). We recently provided genetic evidence supporting the concept that alteration in the ER folding network may be part of the etiology of the disease. Targeted sequencing of ALS cases identified point mutations in two protein disulfide isomerases (PDI) family members, ERp57 (also known as Grp58 or PDIA3) and PDIA1 (also termed PDI) (Gonzalez-Perez et al., 2015). Functional studies indicated that perturbation in the activity of these foldases alters neuromuscular junction structure and function, possibly involving abnormal synthesis of synaptic proteins (Woehlbier et al., 2016). These studies suggested that disrupted ER folding capacity underlays early ALS stages that result in muscle denervation.

## IRE1 Signaling in ALS

The rise of iPSC technology enabled the study of patient motoneurons expressing mutant proteins at endogenous levels (Matus et al., 2014; de Boer and Eggan, 2015). An elegant study using iPSC-derived patient motoneurons harboring a SOD1 mutation discovered that basal physiological levels of ER stress is an intrinsic property of motoneurons linked to their electrical activity (Kiskinis et al., 2014). Furthermore, the exacerbated ER stress in patient motoneurons leads to hyperexcitability, and knock down of XBP1s affords significant neuroprotection (Kiskinis et al., 2014). This concept was also corroborated in motoneurons from patients carrying repeat expansions in c9orf72 and VCP mutations (Kiskinis et al., 2014; Dafinca et al., 2016; Hall et al., 2017), evidencing the transversal role of ER stress in disease etiology. Indeed, we described abnormaly higher levels of the UPR transcription factors ATF4 and XBP1s in post-mortem tissue of sALS cases. To determine the significance of the UPR to ALS pathogenesis *in vivo*, our group performed genetic manipulation of XBP1 or ATF4 in mutant SOD1 mice to assess the functional impact on disease course (Hetz et al., 2009; Matus et al., 2013). Genetic ablation of ATF4 increased the lifespan of mutant SOD1 mice possibly due to attenuated expression of the apoptotic factors CHOP and BIM (Matus et al., 2013). On the other hand, conditional deletion of XBP1s in the nervous system delayed disease onset and extended lifespan of mutant SOD1 mice due to a homeostatic link between the UPR and the autophagy pathway (Hetz et al., 2009). We found that upon ablation of XBP1 expression in mutant SOD1 mice, motoneurons up-regulated the autophagy pathway thus boosting degradation of toxic SOD1 aggregates and slowing disease progression (Hetz et al., 2009). This unexpected effect revealed that the proteostatic networks could be reprogramed through homeostatic responses with potential therapeutic benefits. Using pharmacological approach, we and others provided evidence suggesting that activation of autophagy with pharmacological agents reduce mutant SOD1 and TDP43 aggregates, increasing healthspan (Castillo et al., 2013; Zhang et al., 2014).

The IRE1-XBP1 axis is proposed to play a role in other diseases as well. For instance, we found that deletion of XBP1 in transgenic models of Huntington’s disease (HD) also leads to neuroprotection associated to autophagy induction, enhancing degradation of mutant huntingtin (Vidal et al., 2012). Recently, we have showed that phosphorylation of IRE1 directly correlates with Alzheimer’s disease (AD) progression using histological analysis of post-mortem human brain tissue (Duran-Aniotz et al., 2017). Remarkably, we discovered that activation of IRE1 exacerbates AD pathology by enhancing amyloid precursor protein (APP) expression (Duran-Aniotz et al., 2017). The specific deletion of the RNase domain of IRE1 strongly decreased the deposition of amyloid plaques and neuroinflammation on an AD mouse model. These effects resulted in improved neuronal plasticity and cognitive function (Duran-Aniotz et al., 2017). IRE1 activation also enhances the progression of glioma and its signaling inhibition strongly reduces the growth of tumors *in vivo* (Obacz et al., 2017). Similar findings were reported in other forms of cancer, motivating the screening of small molecules that inhibit IRE1 activity for disease treatment (Hetz et al., 2013; see below). In contrast, many other studies using gain of function to deliver XBP1s into the nervous system using gene therapy revealed important neuroprotective effects in various disease models due to artifical enforcement of adaptive programs governed by the UPR (reviewed in Valenzuela et al., 2016). A complex scenario is emerging where the IRE1-XBP1 pathway can play physiological roles in the nervous system related to neuronal function and plasticity (Martínez et al., 2016) and promote distinct outcomes under pathological conditions depending on the neurodegenerative insult.

The participation of IRE1 signaling in motoneurons demise in ALS has been reported in mutant SOD1 mice (Nishitoh et al., 2008; Lee et al., 2016). Ichijo’s group showed that mutant SOD1 contributes to ER stress by blocking ERAD through a specific interaction with Derlin-1, an ER membrane protein participating in the retro-translocation machinery (Nishitoh et al., 2008). Mutant SOD1 possibly alters Derlin-1 function, leading to impaired ERAD, resulting as a consequence in ER stress and activation of the IRE1-ASK1 apoptotic module. Remarkably, genetic deletion of ASK1 spared motoneurons and delayed disease progression in mutant SOD1 mice (Nishitoh et al., 2008). A recent study extended the role of IRE1 in ALS by identifying homeodomain interacting protein kinase 2 (HIPK2) as an essential component of the IRE1-ASK1 apoptotic cascade, leading to JNK activation under ER stress (Lee et al., 2016). Targeting HIPK2 in mutant SOD1 mice delayed disease onset and prolonged survival due to attenuated motoneuron loss (Lee et al., 2016). Relevantly, activation of HIPK2 positively correlated with TDP-43 proteinopathy in familial C9orf72 and sporadic ALS cases, implicating a broad role of ER stress and IRE1 signaling in ALS pathogenesis (Lee et al., 2016).

## Targeting IRE1 to Treat ALS?

The current state of the field supports a pathogenic role of IRE1 pathway in ALS through activation of cell death programs, besides the possible effects of XBP1s deficiency in enhancing the activity of the autophagy pathway whereas reducing hyperexcitability in motoneurons (Nishitoh et al., 2008; Hetz et al., 2009; Kiskinis et al., 2014; Lee et al., 2016). Moreover, we reason that the sustained activation of IRE1 under chronic ER stress in motoneurons may also lead to exacerbated RIDD through induction of high-order oligomers, potentially causing excessive degradation of mRNA and down-regulation of essential components necessary to sustain motoneuron homeostasis. Indeed, the discovery of ALS genes involved in transcriptional regulation highlighted altered RNA metabolism as a relevant disease mechanism (Peters et al., 2015). In the context of ALS, IRE1 can be envisioned as a hub for integrating proteostasis and RNA metabolism at organelle level, underlying neurodegenerative cascades when these major cellular systems are significantly compromised. It is also conceivable that the deleterious activity of IRE1 may extend beyond motoneurons by enhancing astro- and microgliosis, thus promoting neuroinflammation (Boillée et al., 2006). In fact, the activity of IRE1 has been shown to enhance immune responses, promoting cytokine production in macrophages, in addition to modulate the activity of dendritic cells (Martinon and Glimcher, 2011).

**Figure 2:**
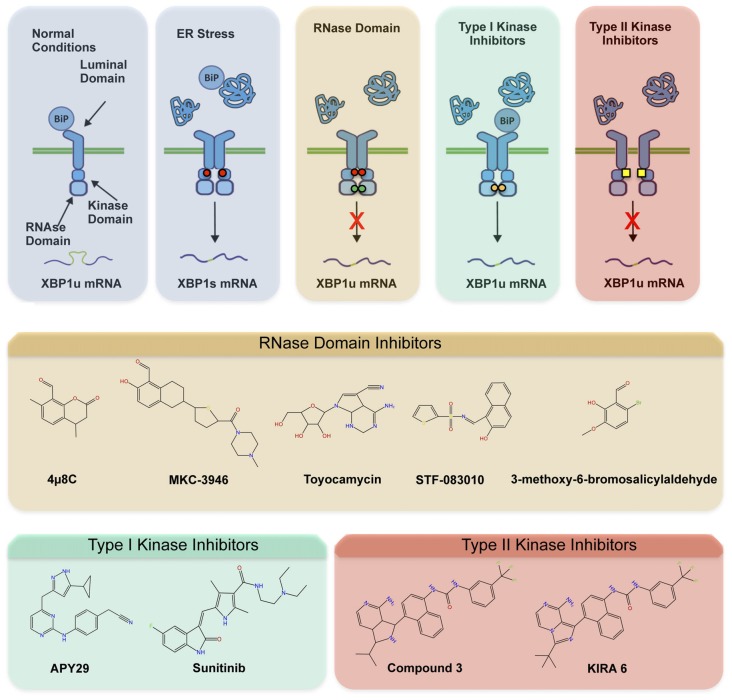
Pharmacological modulation of IRE1. Different classes of inhibitors target distinct IRE1 domains and differentially modulate RNase activity and oligomeric states. **(A)** In the first step of activation, IRE1 suffers dimerization and auto-transphosphorylation to activate its RNase activity and initiate the unconventional splicing of XBP1, in addition to low RIDD activity. Subsequently, IRE1 can form high-order oligomers to potentiate RIDD and catalyze degradation of a select pool of mRNAs and microRNAs. The compounds directly targeting the RNase domain (e.g., 4μ8c, MKC-3946, STF-083110) completely inhibit XBP1 splicing and RIDD without interfering with kinase activity or oligomeric states. The Type I kinase inhibitors (APY29, Sunitinib) prevent auto-transphosphorylation but promote RNase activity and oligomerization by generating conformational changes. The Type II inhibitors (Kinase Inhibitors RNase Attenuators, KIRAs) affect both kinase and RNase activities, possibly altering oligomerization through allosteric interactions. **(B)** Chemical structure of IRE1 inhibitors by mode of action is presented.

The involvement of ER stress and IRE1 signaling in a myriad of diseases has fostered the development of different categories of compounds targeting IRE1 (Figure 2A; Hetz et al., 2013; Maly and Papa, 2014). Most IRE1 inhibitors target the activity of the RNase domain, including 4μ8c, MKC-3946 and STF-083110 (Hetz et al., 2013; Figure 2B). Such compounds indiscriminately shut off XBP1 splicing and RIDD activity, offering an interesting approach where complete inhibition of IRE1 pathway is desired. As suggested by many studies, the abrogation of IRE1 activity in CNS may probably not be the optimal intervention since basal levels of XBP1s appear to be needed for motoneuron homeostasis (Kiskinis et al., 2014), in addition to neuronal plasticity involved in memory and learning-associated processes (Martínez et al., 2016). The recent development of highly potent monoselective IRE1 inhibitors termed *Kinase Inhibitors RNase Attenuators* (KIRA) brings about the possibility to fine-tune IRE1 activity *in vivo* (Ghosh et al., 2014). KIRA compounds act by breaking down IRE1 oligomers thus preferentially blocking terminal IRE1 RIDD activity over XBP1 splicing depending on the dose used (Figures 2A,B). Importantly, KIRA6 has proven efficacious in preserving photoreceptor viability in ER stress-induced retinal degeneration and sustain pancreatic β-cell function in the aggressive Akita diabetic model (Ghosh et al., 2014). More recently, KIRAs were shown to provide protection in models of autoimmune diabetes (Morita et al., 2017). KIRAs did not trigger any evident side effects after systemic administration. It is proposed that KIRAs may specifically target distinct oligomerization states of IRE1, which may selectively affect pathological levels of RIDD triggered by hyperactive IRE1, allowing the signaling through the beneficial effects driven by XBP1s. The optimized use of KIRA compounds in ALS is expected to prevent or halt neurodegeneration by blunting terminal UPR in motoneurons, shifting deleterious RIDD activity to attenuated XBP1 splicing at levels contributing to homeostasis.

In addition to pathology, the UPR has essential roles in various organs, highlighting specialized secretory cells that require a developed ER for their proper function (Cornejo et al., 2013). This is why serious adverse side effects are predicted of the systemic and long-term administration of UPR-targeting drugs (Dufey et al., 2014). However, the recent generation of a conditional knockout mouse for IRE1 in the nervous system demonstrated that it is devoid of any gross spontaneous phenotype. Similarly, the full deletion of IRE1, except in the placenta, generates a viable animal with minor defects in secretory organs (Iwawaki et al., 2009, 2010). Taken together, these observations suggest that blocking the pathway in neurons with small molecules may be safe.

In summary, the compelling evidence in the literature linking ER stress to ALS pathogenesis and the continuous development of specific small molecules to modulate IRE1 activity may offer the opportunity to investigate novel therapeutic approaches to treat this devastating disease. Further research in mouse models of ALS is needed to address the relative contribution of IRE1 signaling in motoneurons and glia to disease progression, in addition to define its mRNA targets under pathological conditions in the nervous system.

## Author Contributions

DBM performed bibliographical research, and wrote and reviewed the article. JVG and PF performed bibliographical research, prepared figures and reviewed the article. CH performed bibliographical research and wrote and reviewed the article.

## Conflict of Interest Statement

The authors declare that the research was conducted in the absence of any commercial or financial relationships that could be construed as a potential conflict of interest.
